# Exploring the surge current degradation of natural ester oil-based nanofluids

**DOI:** 10.1038/s41598-024-57575-0

**Published:** 2024-03-30

**Authors:** Thomas Tsovilis, George Peppas, Evangelos Staikos, Alexandros Hadjicostas, Zacharias Datsios

**Affiliations:** 1 High Voltage Laboratory, School of Electrical and Computer Engineering, Aristotle University of Thessaloniki, 54124 Thessaloniki, Greece; 2 School of Electrical and Computer Engineering, Technical University of Crete, 73100 Chania, Greece

**Keywords:** Electrical and electronic engineering, Energy infrastructure, Fluids

## Abstract

The surge endurance of natural ester oil-based nanofluids against surge events is investigated experimentally. The focus of this work is the examination, through dielectric spectroscopy measurements, of the alteration of the real and imaginary parts of the complex relative permittivity of iron oxide nanofluids as a result of an accelerated degradation test employing a sequence of repetitive current impulses produced via a 12 kV/6 kA combination wave generator. The target is the exploration of a possible implementation of nanofluids as multipurpose liquids that act, in addition to insulation and coolants, as surge absorption media. Promising experimental results are discussed and compared with those of mineral oil that is widely used as a conventional insulating liquid in power transformers.

## Introduction

Nanofluids show a versatile spectrum of applications since they exhibit an enhancement of physicochemical and electrothermal properties of the base liquid^1–4^. Actually, the integration of nanoparticles of different shapes, sizes, and chemical compositions results in increased breakdown voltage^5,6^, lower partial discharge activity^7^, improved heat transfer^8^, and radiation-proof performance^9^ through mechanisms that remain the subject of ongoing investigations. In light of this evidence, natural ester oil-based nanofluids are considered as an environmentally friendly alternative to mineral oils, which are the widely used insulating media and coolants for power transformers^10^.

Currently, the large-scale use of nanofluids in the power industry is impeded by concerns associated with the long-term stability against agglomeration of nanoparticles and consequent sedimentation, as well as the aging due to thermal stress and oxidation^10^. Thus, a series of experimental and theoretical investigations focus on the optimal concentration of nanoparticles in base liquids so as to achieve the advantageous properties of nanofluids that will be maintained for the expected lifetime of the equipment that the liquids will be integrated into^11^.

Although it is well recognized the risk of liquid insulation breakdown due to impinging lightning-related overvoltages^12,13^, the energy absorption capability of nanofluids is sparsely reported and the endurance under impulse currents has not been evaluated. This work introduces an experimental setup for an accelerated surge degradation test that involves a sequence of repetitive high-voltage and high-current impulses. This setup differs from conventional multi-stage impulse voltage generators for the determination of the dielectric strength of liquids that produce impulse currents of limited amplitude and charge^14,15^ which are well below the field experience from lightning electromagnetic pulses; the latter exhibit peak current of several kiloamperes and duration of decades of microseconds^16^. Thus, for the first time with the aid of a combination wave generator and surge-proof test cell, the aging effects of excessive surge current stress are explored by sensing the changes in the complex relative permittivity through dielectric spectroscopy measurements.

Following the global trend for environmentally friendly insulating liquids, that also exhibit low fire hazard and high moisture resistance^17^, this work investigates the surge endurance of natural ester oil-based nanofluids, which are eco-friendly thanks to the biodegradability and nontoxic properties of the vegetable-produced base liquid.

The experimental results of the present study indicate an improved performance of iron oxide nanofluids in terms of surge endurance when compared to natural ester oil base liquid; a discussion is made on the effect of nanoparticles concentration and a comparison is made to surge degradation of mineral oil that is widely used as a conventional insulating liquid in power transformers. This work opens the path for the possible implementation of nanofluids as multipurpose liquid media^18,19^ with surge absorption capability that may result in various applications in the power industry.

## Experimental arrangements

### Sample preparation

The base liquid used for nanofluid preparation is a vegetable-based liquid, Envirotemp FR3^TM^. FR3 shows excellent electrothermal properties^20^ and constitutes an eco-friendly liquid insulation and coolant that has been used for over 20 years in power transformers. The fluid is non-toxic, as evidenced by acute aquatic and oral toxicity assessments, and exhibits high flash/fire points of ~330/360 °C.

**Figure 1:**
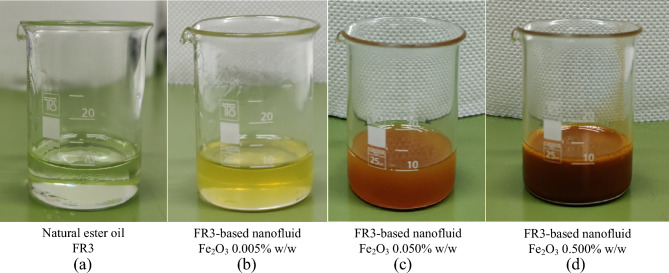
Natural ester oil-based nanofluids of different iron oxide nanoparticle concentrations. (**a**) Natural ester oil FR3, (**b**) FR3-based nanofluid Fe_2_O_3_ 0.005% w/w, (**c**) FR3-based nanofluid Fe_2_O_3_ 0.050% w/w, and (**d**) FR3-based nanofluid Fe_2_O_3_ 0.500% w/w.

Iron oxide (Fe_2_O_3_) nanoparticles with a diameter of less than 50 nm, were incorporated into the natural ester oil (FR3). Three concentrations of FR3-based nanofluids, namely 0.005% w/w, 0.05% w/w, and 0.5% w/w, were prepared as illustrated in Fig. 1. It is noted that the nanofluids exhibit a change in color towards red-brown as the concentration increases; direct visual characterization based on international standards is not applicable. The appropriate quantity of nanoparticles was accurately weighed using a 4-digit scale (Mettler Toledo AB204-S) and added to the natural ester oil by using a 4-hand AtmosBag (Z555525-1EA). Following the integration of nanoparticles into the base liquid, ultrasonication for 4 h was employed with the aid of an ultrasonic cleaner (Branson 1510) to disperse the nanoparticles and homogenize the nanofluids.

Iron oxide nanofluids have been used in the present study in light of the related literature indicating excellent stability and dielectric properties^4,21,22^; low-medium nanoparticle concentrations are related to optimal dielectric strength^23^ and the upper limit refers to unexplored areas with concentrations that resemble those of metal-oxide additives employed in solid-state semiconductors used in the surge protection industry^24^. Exploring the surge endurance of nanofluids with the used nanoparticle concentrations that differ significantly enables the examination of macroscopic effects on surge degradation and establishes a foundation for subsequent investigations.

### Dielectric spectroscopy measurements

**Figure 2:**
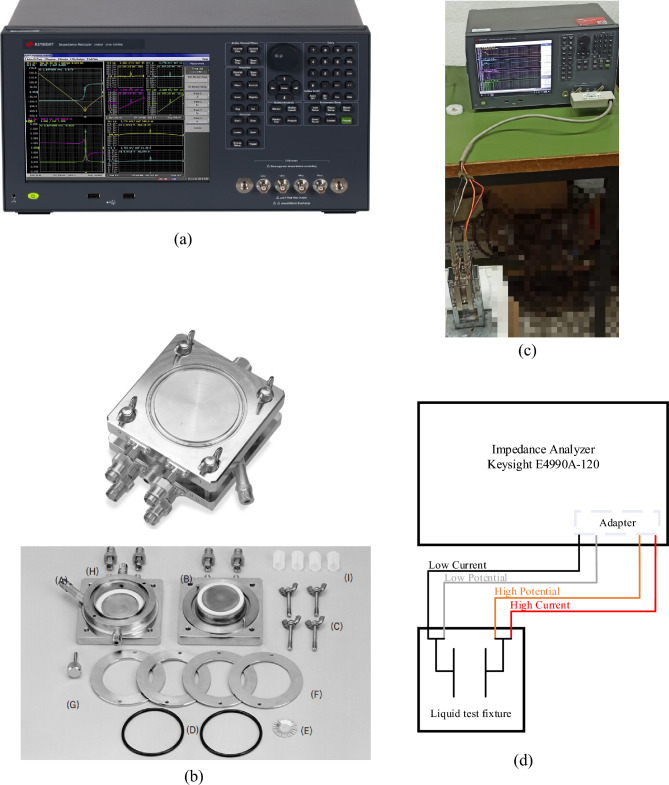
Equipment and experimental setup for dielectric spectroscopy measurements. (**a**) Impedance analyzer, (**b**) liquid test fixture, (**c**) experimental setup, (**d**) schematic diagram.

The prepared nanofluids were inserted into a fixture suitable for liquids’ characterization (Keysight 16452A) and the real and imaginary parts of the complex relative permittivity were determined using an Impedance Analyzer (Keysight E4990A-120) operating at 0.5 V, as shown in Fig. 2.

The dielectric spectroscopy measurements were conducted for a wide frequency range, specifically 50 Hz–5 MHz, covering at the low end (50–400 Hz) the operating frequency of the vast majority of power systems and at the high-frequency domain (10 kHz–5 MHz) the frequencies of transients associated with a wide variety of electromagnetic pulses due to natural (lightning^25^) and artificial (switching operations^26^, NEMP^27^) surge events.

The room temperature was maintained constant at 21 °C and the absolute humidity varied between 15–17 g/m^3^ during the measurements; we followed the test protocol implemented in a recent study^28^.

### Accelerated degradation test

**Figure 3:**
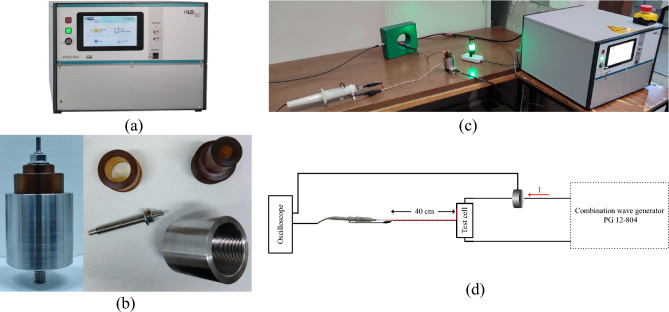
Equipment and experimental setup for accelerated degradation test. (**a**) Combination wave generator, (**b**) custom made test cell, (**c**) experimental setup and (**d**) schematic diagram.

A surge endurance test, simulating accelerated degradation, was conducted to assess the resilience of the natural ester oil-based nanofluids against a sequence of repetitive impulse currents employing the equipment and experimental arrangement depicted in Fig. 3.

**Figure 4:**
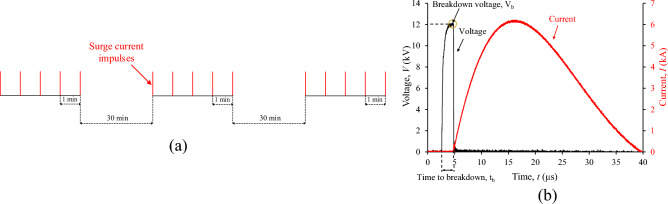
Surge degradation test sequence. (**a**) Sequence of surge current impulses, (**b**) typical impulse voltage and current records.

The impulse current was generated with the aid of a 12 kV/6 kA combination wave generator (HILO PG12-804), following UL 1449^29^ for low-voltage surge protective devices. At each applied impulse voltage of 12 kV, 1.2/50 μs, a breakdown occurs in the micro gap of the test cell as indicated by the collapse of the voltage, and an impulse current of 6 kA, 8/20 μs is conducted as shown in the oscillogram of Fig. 4; it is noted that the impulse current is practically unaffected by the instantaneous breakdown voltage, *V*_*b*_.

The accelerated degradation test sequence involves fifteen exponential current impulses to the specially designed metallic test cell that withstands the excessive pressure associated with the shock wave of supersonic propagation of streamer^30^ and the post-breakdown discharge current conducted through the integrated liquid medium of 10 ml in volume. Three sets of five surges of current impulses were applied with a time interval of 30 min between them^29^; successive current impulses within each group were fixed at 1 min.

After the entire sequence of repetitive current impulses, the degraded liquid from the test cell was used to fill the test fixture and enable the dielectric spectroscopy measurements (Fig. 2).

## Experimental results

**Figure 5:**
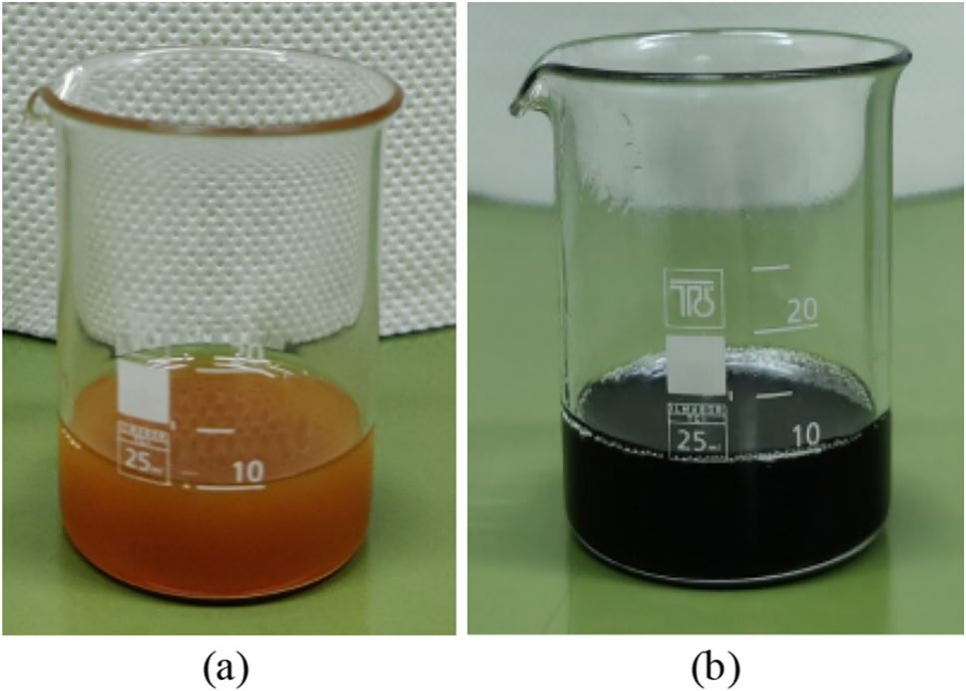
(**a**) Untested (virgin) natural ester oil-based nanofluid and (**b**) degraded nanofluid; iron oxide nanoparticle concentration (0.050% w/w).

The accelerated degradation test results in color darkening that is indicative of the aging of the liquids^31^; degraded nanofluids are nontransparent with a black color depicted in Fig. 5. The changes in complex relative permittivity, ${\upvarepsilon }_{r}^{*}$, which is formulated as the sum of real and imaginary parts as shown in Eq. (1), were obtained through dielectric spectroscopy measurements, and will be presented in what follows.

1 $$\varepsilon_{r}^{*} = \varepsilon^{\prime}_{r} - j\varepsilon^{\prime\prime}_{r} .$$

### Real part of relative permittivity

**Figure 6:**
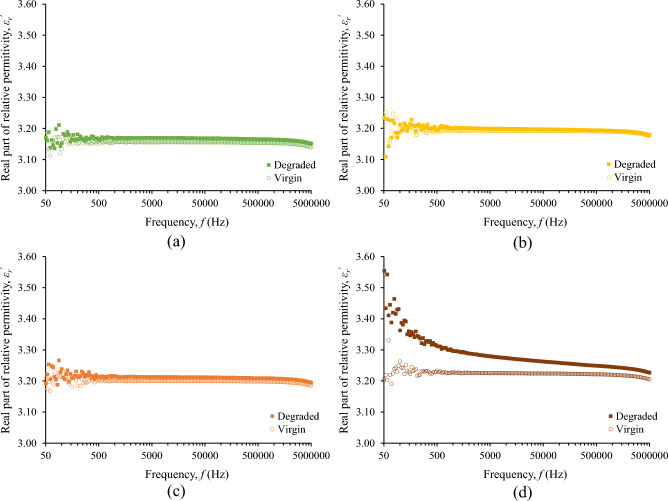
Real part of relative permittivity before and after the accelerated degradation test of natural ester oil-based nanofluids with iron oxides concentration (w/w): (**a**) 0%, (**b**) 0.005%, (**c**) 0.050%, (**d**) 0.500%.

**Figure 7:**
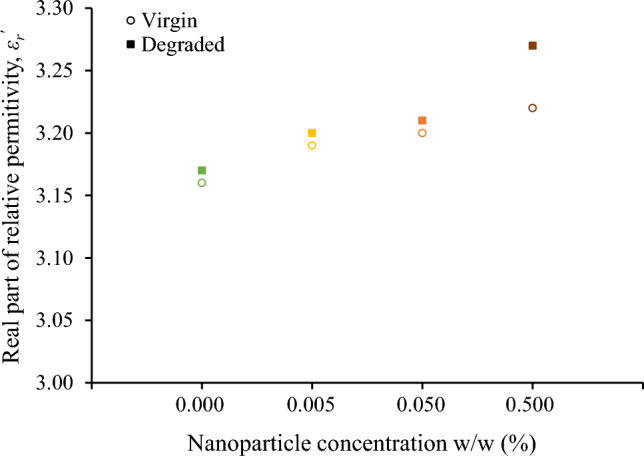
Real part of relative permittivity at 25 kHz for the natural ester oil-based nanofluids under study.

Although the surge degradation of the fluids under study is evident from their color alteration, the change of the real part of relative permittivity, *ε*_*r*_^′^, after the accelerated degradation test is marginal for the base liquid and nanofluids as it can be deduced from Fig. 6. Considering that the *ε*_*r*_^′^ is practically constant for frequencies up to 1 MHz, Fig. 7 illustrates the minimal increase of *ε*_*r*_^′^ for the frequency of 25 kHz, which is of high interest in lightning protection technology^25^, revealing a more marked change for the nanofluid with the higher concentration of iron oxide nanoparticles (0.5% w/w).

### Imaginary part of relative permittivity

**Figure 8:**
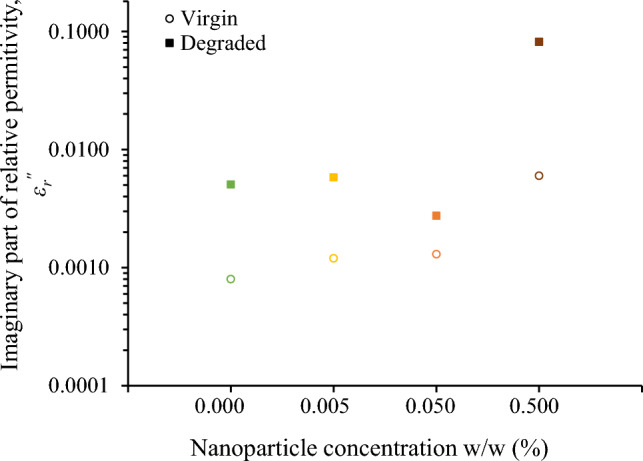
Imaginary part of relative permittivity at 400 Hz for the natural ester oil-based nanofluids under study.

In contrast to the minor alterations observed in the real part of relative permittivity, the imaginary part, *ε*_*r*_^″^, an effective parameter reflecting both polarization losses and conduction losses, undergoes substantial changes due to surge degradation, as depicted in Fig. 8 for the frequency of 400 Hz, which is of high interest in aviation industry.

Thus, *ε*_*r*_^″^ is a key performance indicator revealing the ageing effects associated with the surge degradation test on the electrical conductivity of the fluids, as described by the following equation:

2 $$\sigma = \omega \cdot \varepsilon_{0} \cdot \varepsilon^{\prime\prime}_{r} .$$

**Figure 9:**
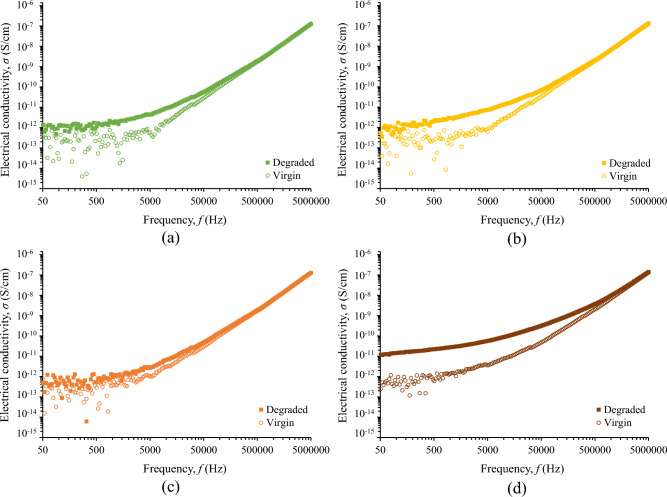
Electrical conductivity before and after the accelerated degradation test of natural ester oil-based nanofluids with iron oxides concentration (w/w): (**a**) 0%, (**b**) 0.005%, (**c**) 0.050%, (**d**) 0.500%.

Figure 9 illustrates the change in electrical conductivity of fluids under study that is more evident at the low-frequency range. This is important since degraded liquids may yield high leakage current in power systems commonly operating at 50/60 Hz. It is noted that the electrical conductivity change is more pronounced for the nanofluid with the higher concentration of iron oxide nanoparticles.

### Discussion on surge endurance of nanofluids and comparison to mineral oil

Fifteen surge current applications (6 kA, 8/20 μs) with a charge transfer of 10 mC/ml each, which is 250 times higher than the charge transfer employed in preliminary experimental investigations of the authors with 2 kA, 1.2/5 μs, that resulted in minor changes in color and impedance of liquid insulation^32^, result in aging of the nanofluids. From Figs. 6 and 9, it can be deduced that for the diagnosis of aging the change of imaginary part of the complex relative permittivity is more effective than the real part.

**Figure 10:**
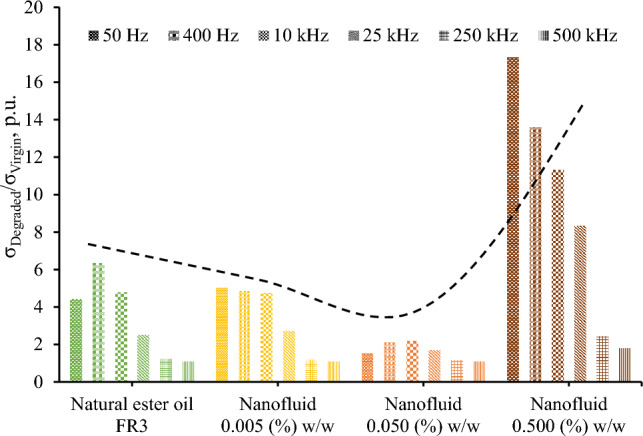
Ratio of electrical conductivity before (*σ*_*Virgin*_) and after (*σ*_*Degraded*_) the accelerated degradation test of natural ester oil-based nanofluids with different iron oxides concentrations; the dotted line illustrates a qualitative general trend.

Figure 10 reveals that the employed severe surge stress resulted in a significant increase of the electrical conductivity of the fluids under study generally of more than two times at 25 kHz; this change is even more pronounced at lower frequencies where most systems operate, such as 50/60 Hz in power and 400 Hz in aviation industry. This low-frequency performance is crucial, considering that leakage current in operating conditions results in power losses and further electrical degradation of insulating fluids. Nevertheless, the electrical conductivity of degraded liquids exhibits an acceptable conductivity of less than 10^–12^ S/cm, for the low-frequency range of 50–400 Hz, as depicted in Fig. 9 with the exception of the nanofluid with the highest iron oxide concentration (0.5% w/w).

It is important to note that the impact of nanoparticle concentration on surge degradation does not exhibit a monotonous trend, with the optimal concentration determined among the investigated levels being 0.05% w/w. A plausible rationale for this observation lies in the hypothesis that medium concentrations of nanoparticles, such as 0.05% w/w, may establish a discharge path for the surge current, thereby mitigating the adverse effects of impulse current stress on the base liquid when compared to natural ester oil and nanofluids with low nanoparticles concentration (0.005% w/w). On the other hand, a further increase in nanoparticle concentration could induce pronounced agglomeration and sedimentation enhanced by surge degradation, leading to a noteworthy increase in electrical conduction through weak links^33^; this hypothesis is supported by visual observation of sedimentation at surge-degraded FR3 fluid. It is noteworthy that this surge degradation performance necessitates additional investigations (i) into the intricate behaviors of nanoparticles under conditions of elevated current conduction along with the mechanisms associated with aging during surge events and (ii) on the long-term stability of nanofluids against agglomeration of nanoparticles.

**Figure 11:**
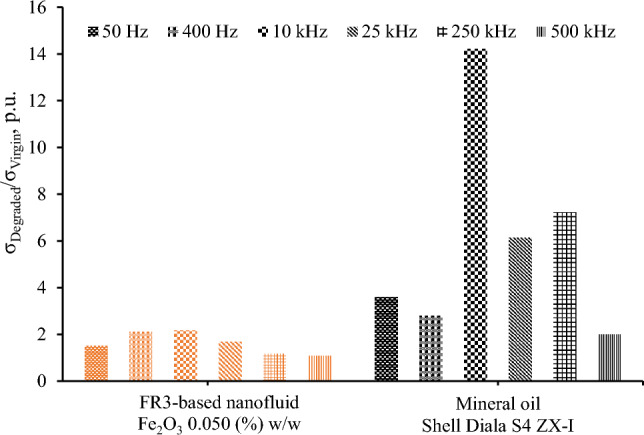
Ratio of electrical conductivity before (*σ*_*Virgin*_) and after (*σ*_*Degraded*_) the accelerated degradation test of FR3-based nanofluid (0.05% w/w) and mineral oil.

Figure 11 illustrates, for comparison purposes, the surge degradation of a mineral oil, Shell Diala S4 ZX-I produced via gas-to-liquid technology, that is commonly employed in the power industry. It is obvious that the surge degradation of mineral oil is 265% higher at 25 kHz than natural ester oil-based nanofluid with medium concentration of nanoparticles (0.05% w/w) which exhibits the highest surge endurance; this is probably associated with the inherent characteristics of the natural ester oil (FR3) and the conductivity of chemical derivatives produced due to the high energy conduction through the different fluids.

The superior surge endurance demonstrated by the FR3-based nanofluid in comparison to mineral oil, coupled with the satisfactory dielectric performance of natural ester oil-based nanofluids^34^ and their fire-resistant properties^17^, establishes a promising framework for the substitution of mineral oils with environmentally friendly liquids facilitated by nanotechnology processes in the forthcoming decades. This prospect, however, necessitates overcoming challenges related to the long-term stability of nanofluids, especially at high nanoparticle concentrations, and addressing aging effects resulting from oxidation and thermal stress^10,35,36^. Available synthesis methods and nanoparticles’ dispersion techniques^21,22,37^ will make feasible the stability of the surge-proof nanofluids; the shape, size, type, chemical treatment, and moisture content of liquids may be parameters that should be examined by the researchers towards this direction.

Furthermore, the resilience of environmentally friendly nanofluids against surge events opens new horizons for their application as versatile materials, potentially extending to applications such as surge arresters and energy storage systems.

## Concluding remarks

The endurance of natural ester oil-based nanofluids against surge events has been experimentally investigated. The evaluation of nanofluid aging has been conducted by examining alterations in the complex relative permittivity resulting from an accelerated degradation test that involves the application of a series of impulse currents of 6 kA, 8/20 μs, with a charge transfer magnitude of 10 mC/ml. Experimental results have shown that:

- A non-monotonous impact of nanoparticle concentration on the surge degradation of nanofluids exists. Actually, FR3-based nanofluids containing an iron oxide nanoparticle concentration of 0.05% exhibit superior surge endurance in comparison to base liquid, which is a vegetable oil, as well as to nanofluids with lower nanoparticle concentrations. Notably, a further elevated concentration of nanoparticles dispersed in the natural ester oil leads to a deterioration in the surge endurance of the base liquid. These results warrant further investigation and theoretical elucidation to understand the underlying mechanisms governing the observed phenomena as well as the determination of the optimal concentration.
- The encouraging surge endurance exhibited by environmentally friendly nanofluids establishes a promising foundation for the exploration of eco-friendly liquids in a diverse range of applications. This extends beyond their conventional use in power transformers, where mineral oils currently predominate, to encompass potential applications such as surge arresters. The robust performance of green nanofluids under surge conditions positions them as compelling candidates for enhancing the sustainability and resiliency of electrical systems, warranting further consideration and exploration in practical applications.

## Methods

### Materials

The base liquid of nanofluids is the Envirotemp FR3^TM^ with typical dielectric strength shown in Table 1 in standard electrode arrangements. The characteristics of Fe_2_O_3_ nanoparticles are provided in Table 2.

**Table 1:** Typical dielectric strength of FR3. Electrode arrangement for DC tests according to IEC 60156.

Voltage	Standard	Breakdown voltage (kV)
AC	IEC 60156	> 55
DC	N/A	> 50
Positive lighting impulse	IEC 60897	> 45
Negative lightning impulse	ASTM D3300	140

**Table 2:** Properties of Fe_2_O_3_ Nanoparticles.

Nanoparticle size	Surface area	Color	Appearance	Structure	Molecular weight
≤ 50 nm	50–245 m^2^/g	Red-brown to brown	Powder	Crystalline (primarily γ)	159.69 g/mol

### Dielectric spectroscopy measurement procedure

The impedance analyzer, following a typical four-terminal measurement procedure determines the amplitude, |*Z*|, and phase angle, *φ*, of the impedance of the samples injected into the liquid test fixture; the latter is connected through an adapter employing a four-cable system (low/high current, low/high potential) as shown in Fig. 2d.

The liquid test fixture electrode arrangement consists of two parallel circular plates at a distance of 1.3 mm forming a capacitor, which can be represented as a resistance, *R*, and a capacitance, *C*, connected in parallel and calculated from measurement results at each frequency *f* as:

3a $$R = \frac{1}{{\left| {\text{Y}} \right|\cos (\theta )}},$$

3b $$C = \frac{{\left| {\text{Y}} \right|\sin (\theta )}}{2\pi f},$$

where |*Y*| and *θ*, are the amplitude, and phase angle of the admittance that is calculated from the measured |*Z*| and *φ*.

To determine the real, *ε*_*r*_*′*, and imaginary, *ε*_*r*_*″*, parts of the complex relative permittivity of each sample two measurements were performed. In the first measurement the fixture is not filled with any liquid and the capacitance of the air gap, *C*_*0*_, is obtained from Eq. (3b). In the second measurement the fixture is filled with the liquid sample and the resistance, *R*_*L*_, and capacitance, *C*_*L*_, are determined through Eqs. (3a, 3b). Next *ε*_*r*_*′*, and *ε*_*r*_*″*, are calculated as^38^:

4a $$\varepsilon^{\prime}_{r} = a\frac{{C_{L} }}{{C_{0} }},$$

4b $$\varepsilon^{\prime\prime}_{r} = a\frac{1}{{2\pi fR_{L} C_{0} }},$$

where *a* is a correction coefficient that is employed to account for the stray capacitance and the edge effects and is calculated for the specific liquid test fixture as^38^:

5 $$a = \frac{{100\left| {\varepsilon_{rm}^{*} } \right|}}{{97.0442\left| {\varepsilon_{rm}^{*} } \right| + 2.9558}},$$

where ${|\upvarepsilon }_{rm}^{*}|$, is given by:

6 $$\left| {\varepsilon_{rm}^{*} } \right| = \sqrt {\frac{{C_{L}^{2} }}{{C_{0}^{2} }} + \frac{1}{{(2\pi fR_{L} C_{0} )^{2} }}}$$

## Data Availability

The datasets used and/or analyzed during the current study are available from the corresponding author on reasonable request.

## References

[1] Cham saard, W., Fawcett, D., Fung, C. C. *et al*. Synthesis, characterisation and thermo-physical properties of highly stable graphene oxide-based aqueous nanofluids for potential low-temperature direct absorption solar applications. *Sci. Rep.* **11**, 16549 (2021). 10.1038/s41598-021-94406-y PMC8367989 34400658

[2] Raza Shah Naqvi, S. M., Farooq, U., Waqas, H. *et al*. Inspection of thermal jump conditions on nanofluids with nanoparticles and multiple slip effects. *Sci. Rep.* **12**, 5586 (2022). 10.1038/s41598-022-07655-w PMC8979999 35379816

[3] Kosinska A, Balakin BV, Kosinski P (2023). Exploring the use of nanofluids in pump-free systems for solar thermal applications. Sci. Rep..

[4] Jiosseu JL, Jean-Bernard A, Mengata Mengounou G (2023). Statistical analysis of the impact of FeO_3_ and ZnO nanoparticles on the physicochemical and dielectric performance of monoester-based nanofluids. Sci. Rep..

[5] Das AK, Chatterjee S (2018). Impulse performance of synthetic esters based-nanofluid for power transformer. Mater. Res..

[6] Suhaimi NS, Md Din MF, Ishak MT (2020). Systematical study of multi-walled carbon nanotube nanofluids based disposed transformer oil. Sci. Rep..

[7] Cianna, A., Sumathi, S. & Jarin, T. Analysis of properties and statistical study on partial discharge inception voltage using normal and Weibull distributions for vegetable oil-based nanofluids. *Biomass Conv. Bioref.* (2024).

[8] Islam T, Alam MN, Niazai S (2023). Heat generation/absorption effect on natural convective heat transfer in a wavy triangular cavity filled with nanofluid. Sci. Rep..

[9] Maaza M, Khamliche T, Akbari M (2022). A novel approach for engineering efficient nanofluids by radiolysis. Sci. Rep..

[10] Oparanti, S. O., Fofana, I., Jafari, R., & Zarrougui, R. A state-of-the-art review on green nanofluids for transformer insulation, *Journ. Mol. Liq.* **396** (2024).

[11] Karatas, M. & Bicen, Y. Nanoparticles for next-generation transformer insulating fluids: A review, *Ren. Sust. En. Rev.* **167** (2022).

[12] Mikropoulos PN, Tsovilis TE, Koutoula SG (2014). Lightning performance of distribution transformer feeding GSM base station. IEEE Trans. Power Del..

[13] Popov, M., Grcev, L., Hoidalen, H. Kr. *et al*. Investigation of the overvoltage and fast transient phenomena on transformer terminals by taking into account the grounding effects. *IEEE Trans. Ind. Appl.* **51**(6), 5218–5227 (2015).

[14] Schon K. *High Voltage Measurement Techniques: Fundamentals, Measuring, instruments, and Measuring Methods*. 1^st^ edn, (Springer, 2019).

[15] Shill DC, Das AK, Chatterjee S (2022). Lightning impulse breakdown performance of saturated vs unsaturated vegetable oil and their mixtures with mineral oil. Ind. Crop. Prod..

[16] CIGRE W.G. C4.407. *Lightning parameters for engineering applications*. Technical Brochure **549** (2013).

[17] CIGRE WG D1.70 TF 3. *Dielectric performance of insulating liquids for transformers*. Technical Brochure **856** (2021).

[18] Shao JJ, Raidongia K, Koltonow A (2015). Self-assembled two-dimensional nanofluidic proton channels with high thermal stability. Nat. Commun..

[19] Saraswat, M., & Sengwa, R. J. Investigations on the optical, dielectric, electrical, and rheological properties of PEG200/ZnO semiconducting nanofluids for soft matter based technological innovations. *Hybr. Adv*. **5** (2024).

[20] Emara MM, Peppas GD, Tsovilis TE (2023). Thermal and dielectric analysis of an ester-based MgO nanofluid. IEEE Trans. Dielectr. Electr. Insul..

[21] Mann S (2009). Self-assembly and transformation of hybrid nano-objects and nanostructures under equilibrium and non-equilibrium conditions. Nat. Mat..

[22] Sabzi Dizajyekan B, Jafari A, Vafaie-Sefti M (2024). Preparation of stable colloidal dispersion of surface modified Fe_3_O_4_ nanoparticles for magnetic heating applications. Sci. Rep..

[23] Chen Q, Beroual A, Sima W, Sun P (2024). AC and lightning impulse breakdown voltage comparative study of mineral oil-based Fe_3_O_4_, Al_2_O_3_, and TiO_2_ nanofluids. IEEE Trans. Dielectr. Electr. Insul..

[24] Anas S, Mahesh KV, Jobin V (2013). Nanofillers in ZnO based materials: A ‘smart’ technique for developing miniaturized high energy field varistors. J. Mater. Chem. C..

[25] IEC 62305-4. *Protection against lightning - Part 4: Electrical and electronic systems within structures* (2010).

[26] Das, J. C. *Transients in Electrical Systems: Analysis, recognition, and mitigation*. 1^st^ edn, (McGraw Hill, 2010).

[27] Electromagnetic pulse (EMP) protection and resilience guidelines for critical infrastructure and equipment. National Coordinating Center for Communications (NCC), National Cybersecurity and Communications Integration Center Arlington, Virginia, USA, Feb. 2019, Unclassified.

[28] Tsovilis, T. E., Staikos, E. V., Hadjicostas, A. Y. *et al*. Relative permittivity of natural ester oil-based nanofluids with iron oxide nanoparticles. *UPEC 2023* Dublin Ireland, (2023).

[29] UL1449. *Surge Protective Devices*, (2022).

[30] Lundgaard, L. E., Liu, Q., Lesaint, O., & Madshaven. I. Dielectric performance of transformer liquids—Summary of a CIGRE study. *IEEE Electr. Ins. Mag*. **39**, 7–16 (2023).

[31] ASTM D1524-15. *Visual Examination of Used Electrical Insulating Liquids in the Field* (2022).

[32] Tsovilis TE, Hadjicostas AY, Staikos EV (2023). Preliminary results on the surge current withstand capability of natural ester oil.

[33] Küchler, A. *High Voltage Engineering*. 1^st^ edn, (Springer Vieweg Berlin, 2018).

[34] Emara MM, Peppas GD, Pyrgioti EC (2022). Thermal and dielectric performance of ester oil-based pentyl-graphene nanofluids. IEEE Trans. Dielectr. Electr. Insul..

[35] Hassanloo H, Sadeghzadeh S, Ahmadi R (2020). A new approach to dispersing and stabilizing graphene in aqueous nanofluids of enhanced efficiency of energy-systems. Sci. Rep..

[36] Koutras KN, Peppas GD, Tegopoulos SN (2023). Aging impact on relative permittivity, thermal properties and lightning impulse voltage performance of natural ester oil filled with semiconducting nanoparticles. IEEE Trans. Dielectr. Electr. Insul..

[37] Das, S. K., Choi, S. U., Yu, W., & Pradeep, T. *Nanofluids: Science and technology*. (John Wiley & Sons, Inc. December 2007).

[38] Keysight Technologies. *16452A Liquid Test Fixture: Operation and service manual*. 7^th^ edn, (Keysight Technologies International Japan G.KApr. 2023).

